# Biomechanical Study of Load to Failure of a Bone Model After Removal of a 2.7-mm or 3.5-mm Plate

**DOI:** 10.7759/cureus.106580

**Published:** 2026-04-07

**Authors:** Devon R Pekas, Swami Rajaram, Wade von Kleeck, Garret Burks, Peter J Apel, Franco M Coniglione, Cody L Evans, Jesse B Seamon

**Affiliations:** 1 Orthopaedic Surgery, Carilion Clinic, Roanoke, USA; 2 Biomedical Engineering, Virginia Polytechnic Institute and State University, Blacksburg, USA

**Keywords:** bone model, both-bone forearm fracture, clavicle fracture, compression, fracture fixation, hardware removal

## Abstract

Objective

This study aimed to investigate the impact of screw hole size on the mechanical strength of bone models following construct removal.

Methods

A locking plate and screws were applied to 34 cylindrical bone models. Seventeen (50%) models were assigned to receive a six-hole plate with 6 3.5-mm screws, and seventeen (50%) models were assigned to receive a ten-hole plate with 8 2.7-mm screws. The screws were subsequently removed, and each model underwent three-point bending and compression tests until bone model failure. Non-inferiority analysis was performed comparing the maximum failure load of the 3.5-mm construct to the 2.7-mm construct. A non-inferiority limit was set at a 10% reduction in the 2.7-mm bone model mean maximum failure load for both the compression and three-point bending tests.

Results

For three-point bend testing, the six-hole 3.5-mm construct was found to be non-inferior to the eight-hole 2.7-mm construct (2145.56 ± 141.04 N versus 2228.37 ± 174.93 N). For compression testing, the 3.5-mm construct cohort was non-inferior to the 2.7-mm construct cohort (14097.73 ± 686.88 N versus 14782.31 ± 841.95 N).

Conclusion

Our bone model data suggest that the biomechanical strength of bone following 3.5-mm construct removal is non-inferior to the biomechanical strength of bone following 2.7-mm construct removal. When selecting fixation that may be later removed, surgeons should choose plate constructs for open reduction and internal fixation that are suited to the fracture fixation without concern that screw hole size affects the biomechanical strength of the bone after hardware removal.

## Introduction

Forearm fractures and clavicle fractures are common injuries in both children and adults [1]. Starting in adolescence, many of these fractures are treated with open reduction and internal fixation utilizing 2.7-mm and 3.5-mm plates and screw constructs [2]. In the pediatric population, especially, the removal of the plate and screw construct can be necessary, or desired, to prevent future complications or due to hardware prominence, typically on the superior aspect of the clavicle or ulnar side of the forearm [3].

The removal of plate and screw constructs can leave the bone susceptible to refracture, presumably from the stress risers left in the bone after screw removal [4-8]. Recently, Cao et al. [5] reported the incidence of forearm refracture following hardware removal as 7.9%, compared to 0.8% when the plate was retained [7]. Most commonly, surgeons utilize plates with 3.5-mm or 2.7-mm screws, and there is no evidence that differences in screw hole size contribute to refracture rate after hardware removal [9]. There is also a lack of understanding of the impact of different screw hole sizes on the mechanical strength of bones following hardware removal. It is assumed that a decrease in the mechanical strength of the bone following screw removal due to a larger screw size could be expected to result in increased refracture rates. Improving the understanding of the impact of screw size on the mechanical strength of the bone will help better inform surgeon decision-making regarding the selection of appropriate hardware in the treatment of fractures. Specifically, in situations where hardware removal is felt to be likely (prominent implant or pediatric population), some surgeons may choose a construct with a smaller screw diameter construct due to the perceived reduced refracture risk, but this is unknown.

This study aimed to investigate the impact of screw size (3.5 and 2.7 mm) on the mechanical strength of bone models representative of small bones such as the clavicle, radius, or ulna. Specifically, we investigated the bone model's mechanical strength through both three-point bending and compression tests while loading each model to failure. We hypothesized that, due to decreased relative bone loss, bone models with 2.7 mm holes would fail at a higher load than the bone models with 3.5 mm holes in both loading conditions. The objective of this study is to compare the post-removal mechanical strength of bone models following fixation with 2.7-mm versus 3.5-mm screw constructs, based on the premise that larger screw holes may reduce structural integrity by removing a greater proportion of cortical bone.

## Materials and methods

There were 34 cylindrical bone models (Pacific Research Laboratories, Seattle, WA), each with a diameter of 16 mm, cortical thickness of 3 mm, and length of 98 mm, and an ultimate compressive strength of 157 MPa. Seventeen (50%) models were assigned to the 2.7-mm construct group, and seventeen (50%) models were assigned to the 3.5-mm construct group.

For the 2.7-mm construct group, a 10-hole 2.7-mm locking compression plate (DePuy Synthes, Warsaw, IN) was centered at the middle of each model, and holes one through four and seven through ten were marked for drilling with a 2.0-mm drill. Similarly, for the 3.5-mm construct group, a six-hole 3.5 mm locking compression plate (DePuy Synthes, Warsaw, IN) was centered at the middle of each model, and all six holes were marked for drilling with a 2.5-mm drill. The 2.7- and 3.5-mm screws were placed, tightened by hand, and removed. In order to mimic the current standard of care, different numbers of screws were used for the 2.7-mm group (eight screws) versus the 3.5-mm group (six screws).

**Figure 1:**
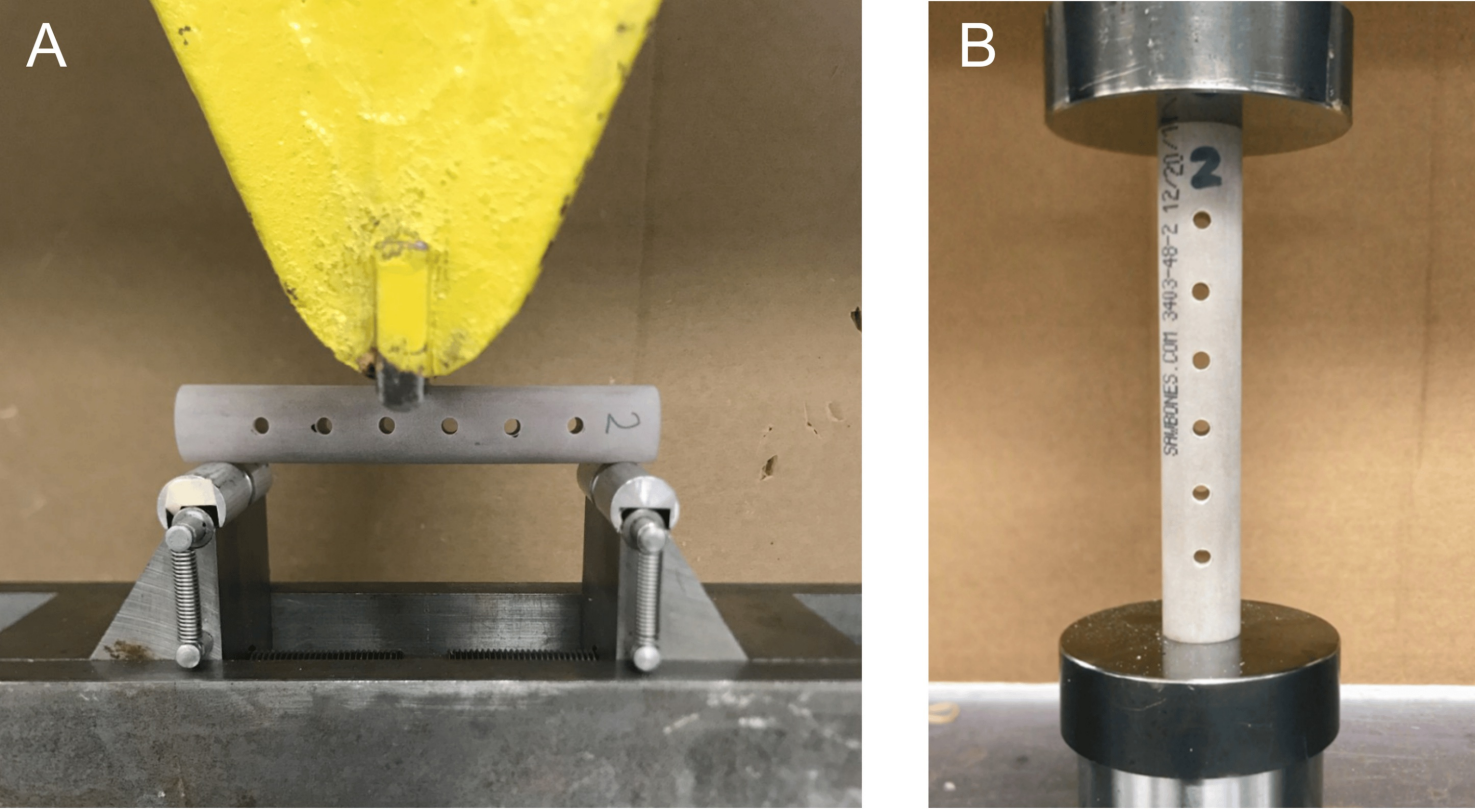
Biomechanical testing setup The universal testing system was configured for the three-point bending test (A) and the axial compression test (B).

For biomechanical testing, three-point bending and compression tests were performed utilizing a universal testing system (Instron, Norwood, MA). For three-point bending tests (Figure 1A), eight (23.53%) models from each group were used. Supports were set at 85 mm wide with the crosshead placed at the center of each model. A transverse preload was applied to the center of each model at a force between 0.002 and 0.005 kN prior to the start of each load-to-failure test. Next, each model was loaded at a constant displacement rate of 0.5 mm/min until bone model failure. The maximum load at which each model failed was collected. For compression testing (Figure 1B), nine (26.47%) models from each group were used, with an axial preload applied to each model of 0.002 to 0.005 kN. Next, each model was loaded to failure at a fixed displacement rate of 0.5 mm/min. The maximum load at which each model failed was collected.

The maximum failure load data were summarized using descriptive statistics. A non-inferiority analysis was performed comparing maximum failure loads between the 2.7-mm construct group and the 3.5-mm construct group using both the three-point bending test and compression test data. A non-inferiority limit was set at a 10% reduction in the 2.7-mm bone model mean maximum failure load for both the compression and three-point bending tests, as a difference that was greater than 10% was judged to be clinically meaningful. The maximum failure load of the bone model with 3.5-mm screws was deemed inferior if the 95% confidence interval (CI) included the non-inferiority limit. A one-sided t-test was performed using the non-inferiority limit and α = 0.025 to determine whether the bone model with 3.5-mm screw holes was statistically inferior in comparison to the bone model with 2.7-mm screw holes.

## Results

**Figure 2:**
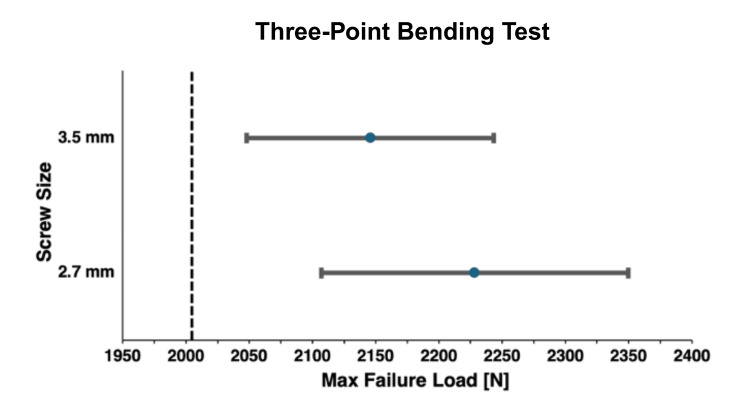
Bone model three-point bending test maximum failure load non-inferiority comparison The maximum failure load mean and 95% confidence intervals for both the 3.5- and 2.7-mm screw sizes are depicted. The non-inferiority limit is displayed as the dotted line, showing that the bone models with 3.5-mm screw holes (N = 8, 23.53%) demonstrated non-inferior failure load compared to the bone models with 2.7-mm screw holes (N = 8, 23.53%) in the three-point bending test. Maximum failure load is represented as mean ± 95% confidence interval (newtons).

For the three-point bending test, the 3.5-mm group failed at a mean load of 2145.56 ± 141.04 N (mean ± SD), while the 2.7-mm group failed at a mean load of 2228.37 ± 174.93 N (Table 1). The absolute difference was 82.81 N with a percent reduction of 3.7%. The 3.5-mm construct cohort was non-inferior (p = 0.987) to the 2.7-mm construct cohort (Figure 2). All models failed at the center, which was the point at which the pressure from the universal testing system was applied.

**Table 1:** Maximum failure load during three-point bend testing *Based on 10% reduction from the 2.7-mm construct maximum failure load. Maximum failure load is represented as mean ± SD (newtons). CI: confidence interval, SD: standard deviation

Screw diameter	Number (%)	Maximum failure load, mean ± SD (newtons)	95% CI
2.7 mm	8 (23.53%)	2228.37 ± 174.93	[2107.15, 2349.58]
3.5 mm	8 (23.53%)	2145.56 ± 141.04	[2047.82, 2243.30]
Non-inferiority margin* (newtons)		t-statistic	p-value
2005.76		2.80	0.987

**Figure 3:**
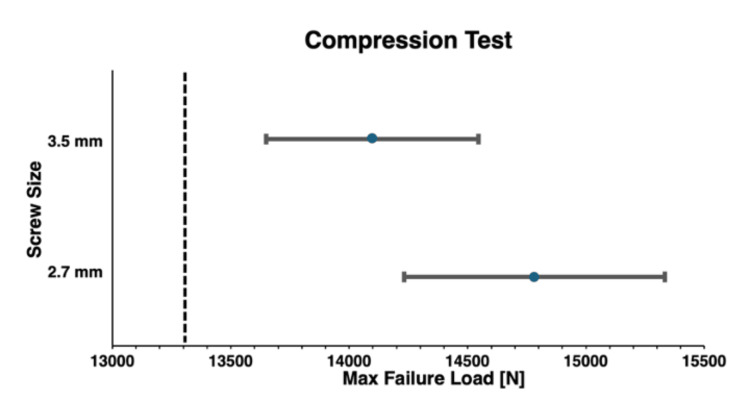
Bone model compression test maximum failure load non-inferiority comparison The maximum failure load mean and 95% confidence intervals for both the 3.5- and 2.7-mm screw sizes are depicted. The non-inferiority limit is displayed as the dotted line, showing that the bone models with 3.5-mm screw holes (N = 9, 26.47%) demonstrated non-inferior failure load compared to the bone models with 2.7-mm screw holes (N = 9, 26.47%) in the compression test. Maximum failure load is represented as mean ± 95% confidence interval (newtons).

For the compression test, the 3.5-mm group failed at a mean load of 14097.73 ± 686.88 N (mean ± SD), while the 2.7-mm group failed at a mean load of 14782.31 ± 841.95 N (Table 2). The absolute difference was 684.58 N with a percent reduction of 4.6%. The 3.5-mm construct cohort was non-inferior (p = 0.996) to the 2.7-mm construct cohort (Figure 3). All models failed at a screw hole located in the lateral half of the bone model.

**Table 2:** Maximum failure load during axial compression test *Based on 10% reduction from the 2.7-mm construct maximum failure load. Maximum failure load is represented as mean ± SD (newtons). CI: confidence interval, SD: standard deviation

Screw diameter	Number	Maximum failure load, mean ± SD (newtons)	95% CI
2.7 mm	9	14782.31 ± 841.95	[14232.24, 15332.38]
3.5 mm	9	14097.73 ± 686.88	[13648.97, 14546.49]
Non-inferiority margin* (newtons)		t-statistic	p-value
13304.08		3.47	0.996

## Discussion

In this biomechanical model of two typical forearm and clavicle fracture constructs, the maximum failure load of the 3.5-mm construct was non-inferior to the maximum failure load of the 2.7-mm construct in three-point bending and axial compression. The majority of forearm refractures occur due to a fall on an outstretched arm [10]. While there is more diversity in the mechanism of clavicle refracture, falls still represent a major mode of failure [11]. Therefore, it is reasonable to believe that these tests are representative of the mechanism that leads to refracture. A subset of refractures occur due to a torsional or combined moment, and future testing may be needed to investigate the impact of torsional failure loads.

The points of failure in our model, which correspond to the sites of hypothesized refracture, were all located in the center for three-point bending. Clinically, this would typically coincide with the location of the old fracture, which has been shown to be the most common site for refracture after pediatric hardware removal [12]. For axial compression, the models failed through one of the lateral screw holes. This supports that screw holes are biomechanically significant stress risers [13], and they are the second most common sites of refracture clinically [12]. These findings may give insight into the types of loads experienced prior to refracture. Given that the different constructs demonstrated non-inferiority in failure loads across both loading conditions, this suggests that other variables beyond the diameter of the screw hole may influence the risk of refracture.

In order to mimic clinical practice and allow easier interpretation of results relative to standard of care forearm and clavicle constructs, different numbers of screws were used for the 2.7-mm group (eight screws) versus the 3.5-mm group (six screws). While the number of screws has been shown not to impact refracture rates after clavicle hardware removal, an increased working length may be protective against refracture [14]. In our case, the 2.7-mm construct had a slightly larger working length due to leaving the middle two holes in the plate unfilled. We suspect that this did not alter our results, as we would have found that the 2.7-mm construct had a superior maximum failure load if this was the case.

Limitations include studying biomechanical properties of physiologic bone using synthetic bone models, which may be unable to replicate cortical bone structure, complex anatomy, biological response, and patient-related variability. In vivo, plate-induced ischemia and stress shielding lead to osteopenia [13], and bones of the forearm subsequently remodel in order to regain pre-injury bone density by approximately six months [15]. Additionally, we did not include a simulated fracture site in the model, as this would have introduced another variable and made the generalizability of the study results more difficult.

## Conclusions

The maximum failure loads after simulated removal of 3.5-mm versus 2.7-mm hardware were non-inferior. Based on the results with bone models, surgeons should consider using plate and screw sizes most appropriate for fracture fixation at the time of open reduction and internal fixation. When hardware removal is anticipated, selection of smaller implants may not be required for the purpose of reducing refracture risk.
